# Tau Inclusions in Alzheimer's, Chronic Traumatic Encephalopathy and Pick's Disease. A Speculation on How Differences in Backbone Polarization Underlie Divergent Pathways of Tau Aggregation

**DOI:** 10.3389/fnins.2019.00488

**Published:** 2019-05-15

**Authors:** Andrzej Stanisław Cieplak

**Affiliations:** ^1^Department of Chemistry, Bilkent University, Ankara, Turkey; ^2^Department of Chemistry, Yale University, New Haven, CT, United States; ^3^Department of Chemistry, Brandeis University, Waltham, MA, United States

**Keywords:** intrinsically disordered proteins, polypeptide backbone, tau aggregation, Alzheimer's disease, Pick's disease, chronic traumatic encephalopathy, aberrant proteostasis, cross-seeding barriers

## Abstract

Tau-related dementias appear to involve specific to each disease aggregation pathways and morphologies of filamentous tau assemblies. To understand etiology of these differences, here we elucidate molecular mechanism of formation of tau PHFs based on the PMO theory of misfolding and aggregation of pleiomorphic proteins associated with neurodegenerative diseases. In this model, fibrillization of tau is initiated by the coupled binding and folding of the MTB domains that yields antiparallel homodimers, in analogy to folding of split inteins. The free energy of binding is minimized when the antiparallel alignment brings about backbone-backbone H-bonding between the MTBD segments of similar “strand” propensities. To assess these propensities, a function of the NMR shielding tensors of the C^α^ atoms is introduced as the *folding potential* function *FP*_*i*_; the C^α^ tensors are obtained by the quantum mechanical modeling of protein secondary structure (GIAO//B3LYP/D95^**^). The calculated *FP*_*i*_ plots show that the “strand” propensities of the MBTD segments, and hence the homodimer's register, can be affected by the relatively small changes in the environment's pH, as a result of protonation of MBTD's conserved histidines. The assembly of the antiparallel tau dimers into granular aggregates and their subsequent conversion into the parallel cross-β structure of paired helical filaments is expected to follow the same path as the previously described fibrillization of Aβ. Consequently, the core structure of the nascent tau fibril is determined by the register of the tau homodimer. This model accounts for the reported differences in (i) fibril-core structure of *in vivo* and *in vitro* filaments, (ii) cross-seeding of isoforms, (iii) effects of reducing/non-reducing conditions, (iv) effects of PHF6 mutations, and (v) homologs' aggregation properties. The proposed model also suggests that in contrast to Alzheimer's and chronic traumatic encephalopathy disease, the assembly of tau prions in Pick's disease would be facilitated by a moderate drop in pH that accompanies e.g., transit in the endosomal system, inflammation response or an ischemic injury.

## Introduction

Aberrant proteostasis appears to be at the core of a host of neurodegenerative and mental disorders, from Alzheimer's to schizophrenia (Bradshaw and Korth, 2018). Thus, a thorough understanding of what makes some proteins prone to aberrant folding may be necessary to meet one of the most pressing challenges of modern times. Unfortunately, our current understanding of protein folding and misfolding is limited for want of a physicochemical theory of protein secondary and tertiary structure (Baldwin and Rose, 1999). Recognizing these limitations, an attempt was recently made to construct such a theory, using the PMO theory-informed approach and focusing on the electronic configuration and hyperconjugation of the peptide amide bonds (Cieplak, 2017). To capture the effect of polarization of peptide linkages on the conformational and H-bonding propensity of the polypeptide backbone, a function of the NMR shielding tensors of the C^α^ atoms was introduced as the *folding potential* function *FP*_*i*_. The *FP*_*i*_ function proved to be an effective tool to investigate conformational behavior of the intrinsically disordered and pleiomorphic proteins, revealing a common pattern of backbone density distribution in the amyloidogenic regions of several highly pleiomorphic proteins associated with neurodegenerative diseases: amyloid beta Aβ, tau, α-synuclein αS, and mammalian prions PrP^C^. A common molecular model of aggregation of these proteins was consequently proposed (Cieplak, 2017).

Here we apply this model to address the complexities of polymerization of tau. There is a growing recognition of the role of tau in a wide range of brain proteinopathies and consequently a growing interest in the structure and the mechanism of self-replication and cell-to-cell transmission of tau prions (Bemporad and Chiti, 2012; del Carmen Cárdenas-Aguayo et al., 2014; Hasegawa, 2016; Wang and Mandelkow, 2016; Goedert and Spillantini, 2017; Goedert et al., 2017; Guo et al., 2017; Nizynski et al., 2017; Ayers et al., 2018; Demaegd et al., 2018; Gao et al., 2018; Sebastién-Serrano et al., 2018). Fibrillization of tau appears broadly similar to the fibrillization of Aβ and αS but relatively few details are available concerning its early stages and the nature of low-order oligomers (Pavlova et al., 2016; Eschmann et al., 2017; Huang et al., 2018). It is believed that fibrillization is initiated by the dimerization of tau (Friedhoff et al., 1998; Sugino et al., 2009; Kumar et al., 2014) which, it is now reported, may involve a host of seeding-competent monomer conformers that encode strains of tau prions (Mirbaha et al., 2018; Sharma et al., 2018). The dimerization is followed by the assembly of dimers into granular aggregates (Maeda et al., 2007; Ren and Sahara, 2013; Karikari et al., 2019) and subsequent conversion of these aggregates into filaments. The process apparently involves divergent paths of dimerization and oligomerization since the tau-related dementias are found to involve specific to each disease aggregation pathways and morphologies of filamentous tau assemblies (Sanders et al., 2014; Dujardin et al., 2018). Thus, the cryo-EM investigation has recently shown that paired helical filaments of tau isolated from the brains of Pick's, Alzheimer's and chronic traumatic encephalopathy (CTE) patients are considerably different in their fibril-core structure (Fitzpatrick et al., 2017; Falcon et al., 2018, 2019), while the *in vitro* heparin-induced fibrillization of the 4R isoform was found to yield a heterogenous mixture of several types of filaments, none in the Pick or Alzheimer fold (Fichou et al., 2018; Kjaergaard et al., 2018; Zhang et al., 2018). The filamentous deposits of tau can also be different in terms of the isoform composition which turns out to be specific to each disease as well. For instance, Pick's filaments contain only 3R isoforms, progressive supranuclear palsy filaments only 4R isoforms, while Alzheimer's filaments contain both 3R and 4R isoforms. The reasons for the presence or absence of barriers to cross-seeding of the 3R and 4R aggregates are not well-understood (Adams et al., 2010; Siddiqua et al., 2012; Yu et al., 2012; Kumar and Udgaonkar, 2018; Weismiller et al., 2018). In this paper, we describe molecular mechanism of fibrillization of tau which attributes the observed diversity of fibril morphology and asymmetric cross-seeding barriers to the variation in the pattern of backbone polarization and the concomitant variation in the conformational and H-bonding propensity of the amyloidogenic region of tau. The hypothesis is based on the investigation of the folding potential *FP*_*i*_ profiles for the MTB domains of tau and a few truncated tau constructs.

### Computational Methods. A Protocol for Evaluation of Secondary Structure Propensity

The *folding potential* function *FP*_*i*_ is a function of the NMR shielding tensors of the C^α^ atoms, **σ**(C^α^)^Xaa^, which were obtained by quantum mechanical modeling of protein secondary structure. The calculations were carried out using the oligopeptides AcGXaaGGGNH_2_ and AcGGGGGXaaNHMe as the models of the 3_10_-helix and the hairpin with the type Ib reverse turn, respectively, at the B3LYP/D95^**^ level of the theory [Gaussian 98, Revisions A.3, A.7, A11.2 (Frisch et al., 1998)], according to the protocol described previously in Cieplak (2017). The canonical and covalently modified residues Xaa (the L-amino acid series) of the hexapeptide hairpin and the pentapeptide helix were systematically varied, taking into account side chain conformations (Kyte, 1995) and ionization state when appropriate, to yield the total of 141 congener structures. To compute the NMR shielding tensors using the atomic coordinates of the obtained structures, the B3LYP/D95^**^ and GIAO (Gauge-Independent Atomic Orbital) methods were employed. The obtained **σ**(C^α^)^Xaa^ tensor values are used to quantify the relationship between the density distribution and the conformational and H-bonding propensity of the polypeptide backbone via construction of *the folding potential* function *FP*_*i*_. The folding constants σ^Xaa^ are first derived from the linear normalization of the mean **σ**(C^α^)^Xaa^ tensor values to the scale where the σ^Pro^ constant for proline is −1 and the σ^Gly^ constant for glycine is 1, see Table 1. The folding potential at the residue *i, FP*_*i*_, is then defined as the averaged sum of the mean μ_*i*_ and standard deviation σ_*i*_ of the constants σ^Xaa^ within the three-(*i*−1, *i, i*+1) and five-(*i*−2, *i*−1, *i, i*+1, *i*+2)-residue windows:

(1) $$\begin{array}{l} FP_{i}=½[{\text{μ}}_{i}\left(\sigma_{\;j}^{\text{Xaa}};j=i-1,i,i+1\right)\\ \;+{\text{σ}}_{i}\left({\text{σ}}_{\;j}^{\text{Xaa}};j=i-1,i,i+1\right)\\ \;+{\text{μ}}_{i}\left({\text{σ}}_{j}^{\text{Xaa}};j=i-2,i-1,i,i+1,i+2\right)\\ \;+{\text{σ}}_{i}\left({\text{σ}}_{\;j}^{\text{Xaa}};j=i-2,i-1,i,i+1,i+2\right)] \end{array}$$

In addition, the slope of the folding potential at the residue *i*, Δ*FP*_*i*−*1*→*i*+*1*_, is approximated by the difference of the folding potential at the residues *i*−*1* and *i*+*1*:

(2) $$\begin{array}{l} \Delta FP_{i-\text{1}\to i+\text{1}}=FP_{\text{i+1}}-FP_{\text{i}-\text{1}} \end{array}$$

**Figure 1:**
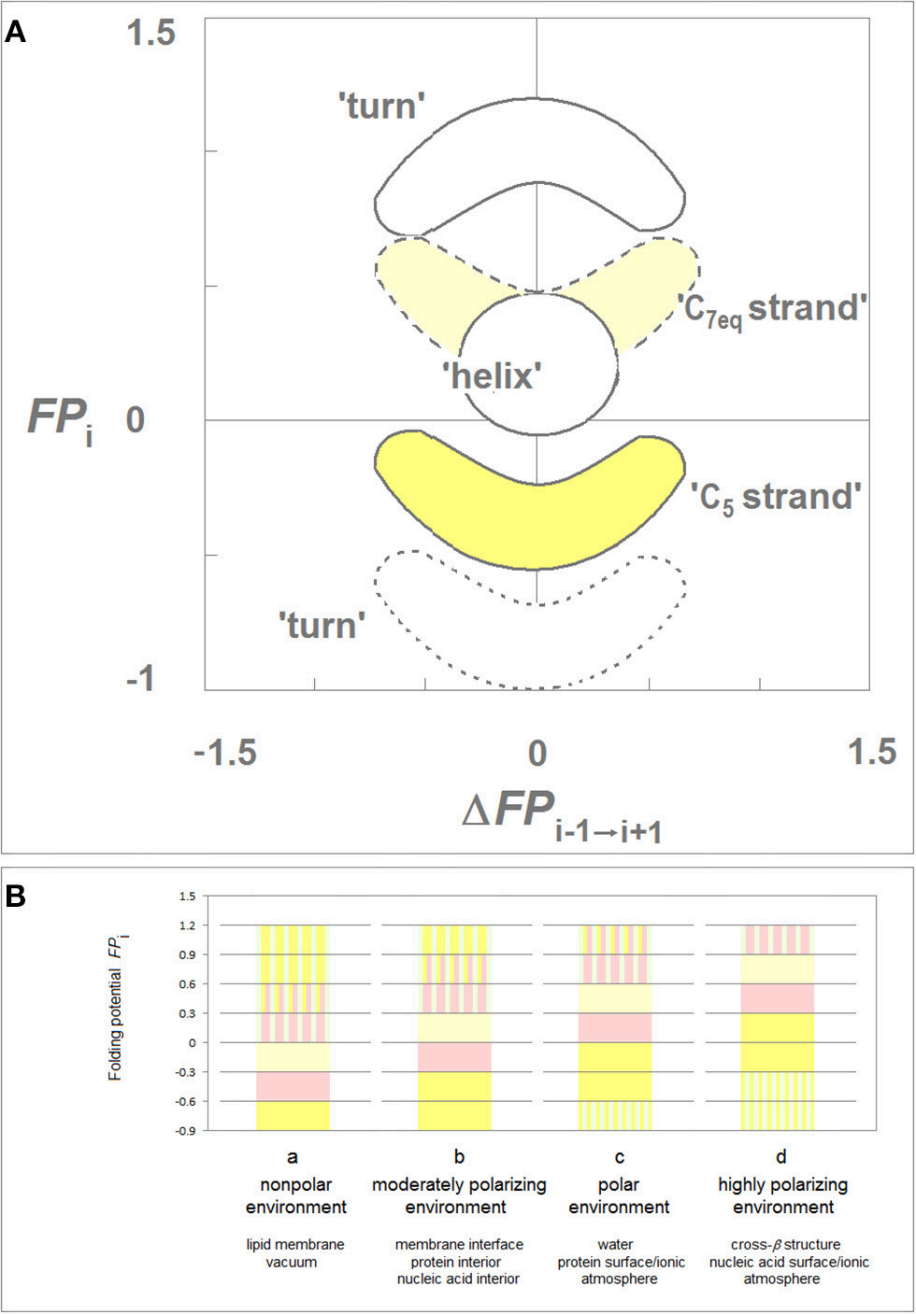
Folding potential function *FP*_*i*_ as a probe of the three-dimensional structure of proteins. **(A)** Characteristic clusters of the data sets in the plots of *FP*_*i*_ vs. the “slope” of *FP*_*i*_, Δ*FP*_*i*−__*1*→*i*+*1*_. The five clusters of data correspond to the three archetypal elements of the secondary structure: e.g., the presence of the archetypal “helix” will be marked by a compact cluster of data sets in the center of the plot. The ordinate of this cluster will vary since the optimal *FP*_*i*_ value for “helix” depends on the medium's capacity to polarize the protein, *vide infra*. Notice that “strand” and “turn” have each two avatars: (i) “C_5_ strand” and “C_7eq_ strand,” and (ii) “*FP*_*i*_ >> 0 turn” (defined here as the three- or five-residue segment that incorporates Gly in the center) and “*FP*_*i*_ < < 0 turn.” **(B)** Folding potential, medium properties and secondary structure preferences of the polypeptide backbone: (a) The *FP*_*i*_ values that ensure stability of the periodic secondary structure in a non-polar environment such as the lipid matrix of the bilayer membrane or vacuum: the optimal *FP*_*i*_ range for the α-helix is −0.6 to −0.3 and the optimal *FP*_*i*_ ranges for β structure is < −0.6 (C_5_ strand) and −0.3 to 0 (C_7eq_ strand). The less polarized segments are malleable in a non-polar aprotic medium and may adopt helical (3_1_-helix, PP_II_-helix, α^*^-helix) folds while the least polarized segments of the polypeptide backbone, e.g., a sequence of consecutive “*FP*_*i*_ >> 0 turns,” may adopt the extended (C^*^_5_ strand) folds depending on molecular embedding; (b) The *FP*_*i*_ values that ensure stability of the periodic secondary structure in a moderately polarizing environment such as the bilayer membrane interface, the interior of a soluble protein globule or the interior of the DNA duplex: the optimal *FP*_*i*_ range for the α-helix is −0.3 to 0 and the optimal *FP*_*i*_ ranges for β structure is < −0.3 (C_5_ strand) and 0 to 0.3 (C_7eq_ strand); (c) The *FP*_*i*_ values that ensure stability of the periodic secondary structure in a polar medium such as the physiological 1:1 electrolyte solution: the range of the optimal *FP*_*i*_ values for the α-helix is now 0 to 0.3 while the somewhat less and more polarized segments are likely to form β-sheets. The most polarized segments are now likely to form “*FP*_*i*_ < < 0 turns” or PP_II_-helix. The sequence of consecutive “*FP*_*i*_ >> 0 turns” forms a random coil in an aqueous buffer unless it is stabilized by molecular embedding in helical (3_1_-helix, PP_II_-helix, α^*^-helix) or extended (C^*^_5_ strand) folds; (d) The *FP*_*i*_ values that ensure stability of the periodic secondary structure in the hypothetical highly polarizing environment such as the pre-organized ionic grid e.g., on the surface of a DNA or RNA strand (the sequence of consecutive “*FP*_*i*_ >> 0 turns” is likely to form here an α^*^-helix), or the microenvironment of the extended β structure of β solenoid or amyloid filament.

While the folding potential function *FP*_*i*_ does not carry any information about the constraints introduced e.g., by the hydrophilic residues or by proline and obviously does not take into account any side chain-side chain interactions, the *FP*_*i*_ and *FP*_*i*_ vs. Δ*FP*_*i*−*1*→*i*+*1*_ plots were found to identify essential elements of the secondary and tertiary structure of proteins when two theories of solutions, the Onsager theory of solute-solvent polarization (Onsager, 1936) and the Debye-Hückel theory of dilute solutions of strong electrolytes (Debye and Hückel, 1923), are taken into account. The main features of this model are summarized in Figure 1 which comprises basic elements of the previously described theory (Cieplak, 2017).

**Table 1:** Folding constants σ^Xaa^ of the canonical amino acids:^a,b^ σ^Xaa^ = {[**σ**(C^α^)^Xaa^(*trans*) + **σ**(C^α^)^Xaa^(–*gauche*)]—[**σ**(C^α^)^Gly^ + **σ**(C^α^)^Pro^]}/[**σ**(C^α^)^Gly^ – **σ**(C^α^)^Pro^].

**Xaa**	****σ**^**Xaa**^**	**Xaa**	****σ**^**Xaa**^**
A	0.1898	K	−0.0772
C	−0.4989	L	−0.0441
C[SMe]	−0.0403	M	−0.2143
D	0.1293	N	0.0296
D^−^	−0.1087	P	−1
E	0.1889	Q	−0.2485
E^−^	−0.4847	R	0.1683
F	−0.4289	S	−0.4700
G	1	T	−0.9066
H	−0.2917	V	−0.7703
H^+^	0.2584	W	−0.2704
I	−0.7647	Y	−0.3981

^a^Each tensor **σ**(C^α^) is the average of the values obtained with two models of secondary structure, a hairpin (AcGGGGGXaaNHMe/Ib) and a helix (AcGXaaGGGNH_2_/3_10_), at the GIAO//B3LYP/D95^**^ level of the theory. The mean values for the trans and –gauche conformers of the side chain about the C^α^-C^β^ bond are taken when appropriate (Kyte, 1995).

^b^*The σ^Xaa^ constants for some covalently modified amino acids are: Ser O^γ^-${\text{PO}}_{\text{3}}^{-\text{2}}$−1.1584, Ser O^γ^-${\text{PO}}_{\text{3}}^{-\text{1}}$ approx. −0.6160, Met S(= O)_2_−0.1101, Lys N^ξ^-COCH_3_−0.2518, Val C^β^-CH_3_−0.9993, Ala C^β^-F_3_−0.4258, Phe -F_5_−0.0829, Leu C^δ^-F_3_/C^δ^-F_3_ 0.0049, Ala C^β^-CF_3_ 0.2713, Ala C^β^-n-CH_2_CH_2_CH_3_−0.2014, Thr C^β^-N^γ^H_2_−0.8477, Gly C^α^-C=N 0.8893, Gly C^α^-C=NO 0.8500, Gly C^α^-C=CH 0.6828*.

**Figure 2:**
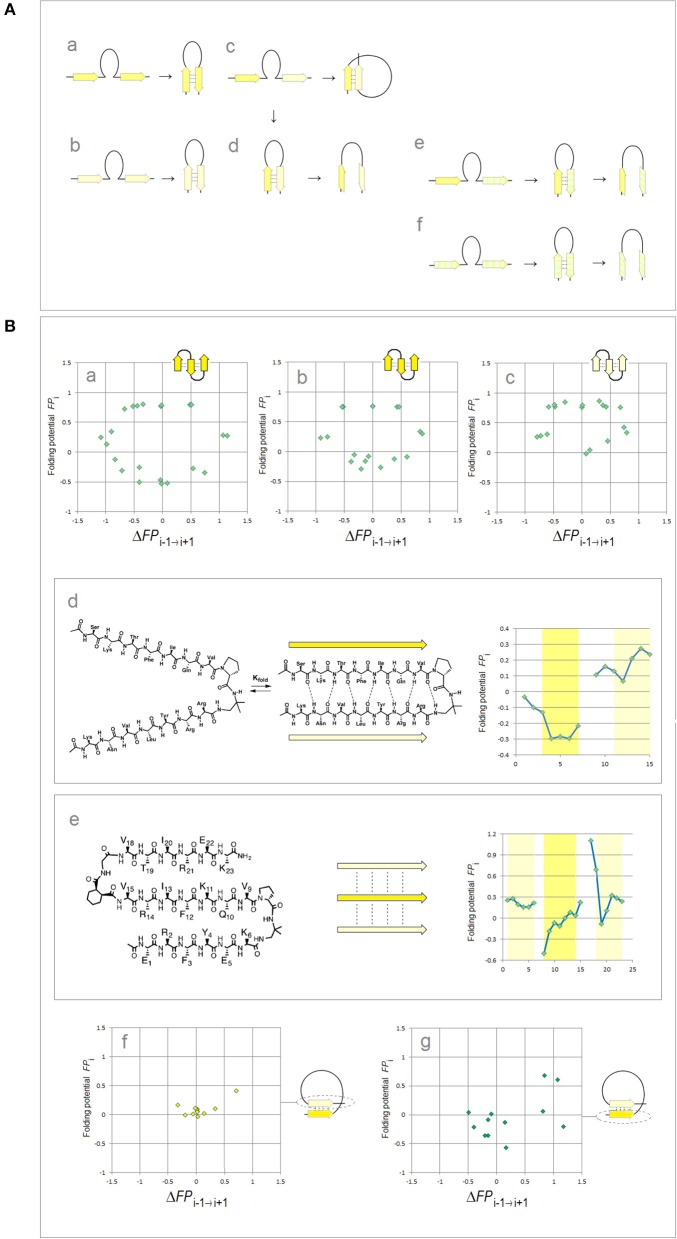
Electronic configuration of the polypeptide backbone and molecular recognition in formation of β structure (Cieplak, 2017). **(A)** A model of alignment preferences in the complexes of the C_5_ and C_7eq_ strands. The polypeptide segments comprising two consecutive strands form stable β-hairpins (antiparallel assembly) when the two strands are either (a) both highly polarized (C_5_↑C_5_↓) or (b) both moderately polarized (C_7eq_↑C_7eq_↓). In contrast, when one strand is highly polarized and the other is moderately polarized, these segments are expected to form (c) β-solenoid coils (parallel assembly, C_7eq_↑C_5_↑) or (d) unstable β-hairpins (antiparallel assembly C_7eq_↑C_5_↓) which are prone to convert into β-arches; similarly when one strand is highly polarized (C_5_) and the other is least-polarized (${\text{C}}_{5}^{*}$) (e), or both strands are least-polarized (f), the segment may form a hairpin which is also prone to convert into β-arch. Such conversions are particularly likely when the backbone H-bonding between the least-polarized strands is relatively weak and when the β-arch-like structure can be stabilized as a “steric zipper.” **(B)** Electronic configuration of the polypeptide backbone in the autonomous β sheets. In the plots of *FP*_*i*_ vs. the “slope” of *FP*_*i*_, Δ*FP*_*i*−__1 → i+1_ (cf. Figure 1), the presence of the archetypal antiparallel “sheet” would be marked by a circular distribution of data sets that combines the “C_5_ strand”/“*FP*_*i*_ >> 0 turn” or “C_7eq_ strand”/“*FP*_*i*_ >> 0 turn” clusters while the presence of the parallel “sheet” would be marked by a combination of the “C_5_ strand” and “C_7eq_ strand” clusters. The *de novo* designed three-stranded antiparallel β-sheets (three-stranded β meanders) (de Alba et al., 1999; Griffiths-Jones and Searle, 2000; Lopez de la Paz et al., 2001) and two- and three-stranded parallel β-sheets (Fisk et al., 2006; Kung et al., 2015), and the two-stranded parallel β-sheets embedded in left-handed coils from the C-terminal domains of the penicillin binding protein PBP2x from *Streptococcus pneumoniae*, PDB ID 1k25, provide examples of the *FP*_*i*_ profiles which are consistent with this model: (a) KGEWTFVNGKYTVSINGKKITVSI, ~50% in β structure, H_2_O, pH 3, 25°C (C_5_↑C_5_↓C_5_↑-meander); (b) TWIQNGSTKWYQNGSTKIYT, 20–30% in β structure, H_2_O, pH 3.25, 10°C (C_5_↑C_5_↓C_5_↑-meander); (c) RGWSLQNGKYTLNGKTMEGR, ~35% in β structure, 10% D_2_O/H_2_O or D_2_O, pH 5, 0–10°C (C_7eq_↑C_7eq_↓C_7eq_↑-meander); (d) C_5_↑C_7eq_↑-parallel sheet, cf. the *FP*_*i*_ plot. The C-termini of two strands are connected by the D-prolyl-1,1-dimethyl-1, 2-diaminoethane unit (diamine linker D-Pro-DADME), ~64% “folding-core” residues (F5-V8 and R11-L14) in β structure at 10°C, 10% D_2_O/H_2_O, 100 mM sodium acetate buffer, pH 3.8; (e) C_7eq_↑C_5_↑C_7eq_↑-parallel sheet, cf. the *FP*_*i*_ plot. The C-termini of strands 1 and 2 are connected by the diamine D-Pro-DADME while the N-termini of strands 2 and 3 are connected by the diacid formed from (1*R*,2*S*)-cyclohexanedicarboxylic acid (CHDA) and Gly, 4°C, 10% D_2_O/H_2_O, 2.5 mM sodium [D_3_]acetate buffer, pH 3.8; (f) the C_7eq_ strands from two C_5_↑C_7eq_↑-parallel sheets in the left-handed coils of PBP2x from *Streptococcus pneumoniae*, PDB ID 1k25; (g) the C_5_ strands from two C_5_↑C_7eq_↑-parallel sheets in the left-handed coils of PBP2x, PDB ID 1k25.

Lastly, binary complexes of oligopeptides (AcAAANHMe)_2_ and (AcAAAAANHMe)_2_ were obtained by unconstrained optimization [as described above, at the B3LYP/6-31G^*^ level of the theory, completed by the default convergence criteria of Gaussian98, cf. structures **6a**–**6d** and **7a**–**7b**/**8a**–**8c**, respectively, in Cieplak (2017)]. The individual strands in these complexes were found to optimize either to the C_5_ or the C_7eq_ (2_7_-ribbon) geometries, and their conformations are same in the antiparallel complexes (C_5_↑C_5_↓ or C_7eq_↑C_7eq_↓) and mixed in the parallel complexes (C_7eq_↑C_5_↑); *the antiparallel complexes with mixed strand conformations (C*_7*eq*_↑*C*_5_↓*) were found to be unstable in unconstrained optimizations*. These findings suggest that the preferred mode of assembly of the two-stranded β-sheets depends on charge polarization of the main chain as well. Accordingly, a mechanism of *FP*_*i*_-directed molecular recognition in formation of β structure is presented in Figure 2 which comprises main elements of the previously described theory (Cieplak, 2017).

## Results and Discussion

### (i) Electronic Configuration of the Polypeptide Backbone and the Antiparallel-to-Parallel β Structure Conversion as a Path to Paired Helical Filaments of Tau

**Figure 3:**
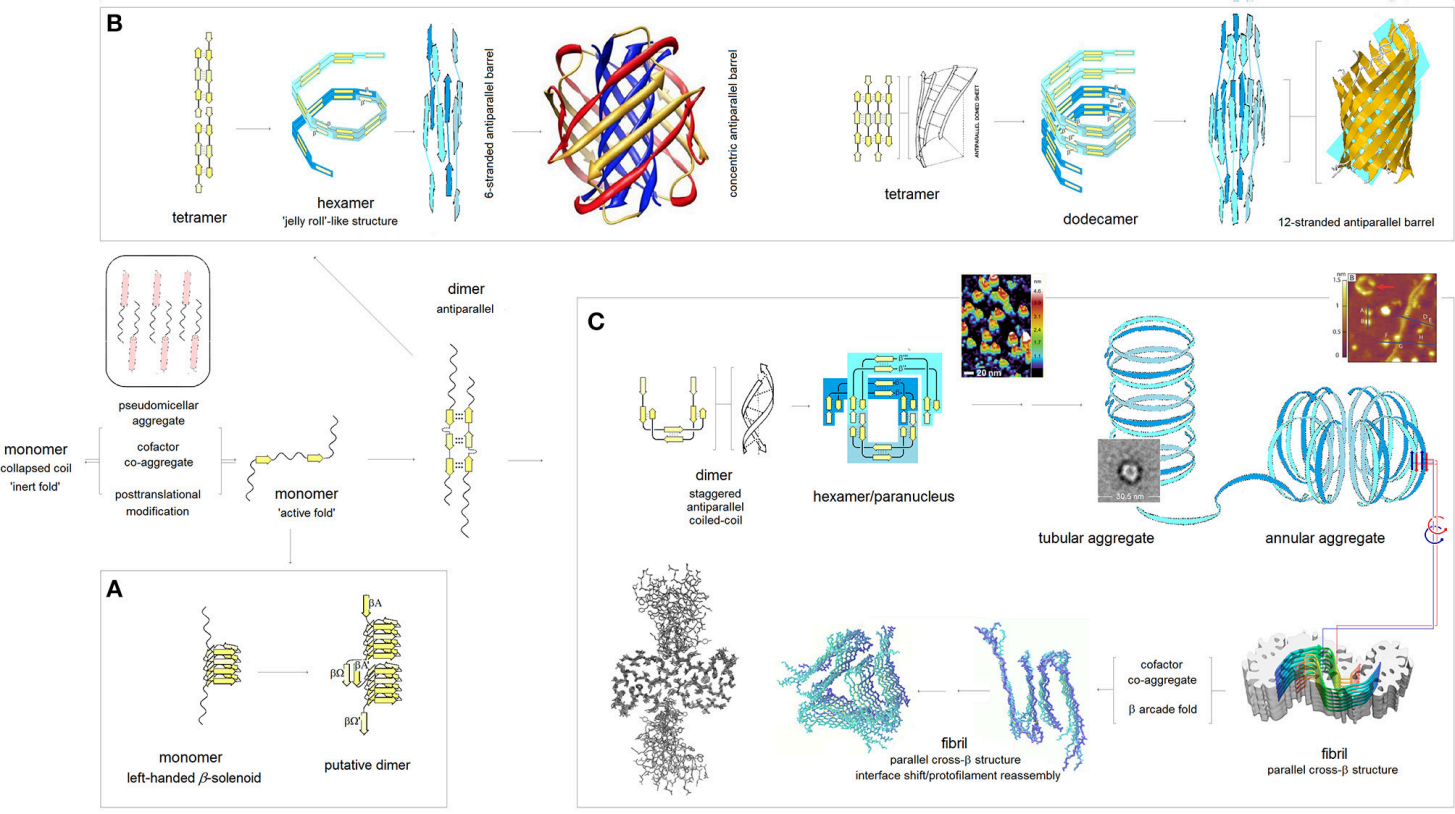
The PMO theory of misfolding and aggregation of pleiomorphic proteins associated with common brain proteinopathies. Based on the PMO theory of protein secondary and tertiary structure (Cieplak, 2017) and conformational properties of β sheets (Salemme, 1983; Branden and Tooze, 1999), polymerization of Aβ, tau, αS and PrP^C^ is initiated by an “active” fold of the intrinsically disordered amyloidogenic region of a monomer. The “gain-of-structure” for the disordered region is realized either by folding into a β solenoid **(A)**, or coupled binding and folding into an antiparallel dimer by analogy to the coupled binding and folding of split inteins, which may lead to (i) antiparallel β barrel structures **(B)**, or (ii) parallel cross-β structures **(C)**. **(A)** Formation of the β solenoid structures: Folding of β solenoids is assumed to require the presence of a sequence of alternating “C_7eq_ strand” and “C_5_ strand” propensities (in the parallel alignment, the free energy of the backbone-backbone H-bonding is minimized, according to the underlying PMO theory, by the binding backbone segments with *contrasting* “strand” propensities e.g., C_7eq_↑C_5_↑, cf. Figure 2), and a sequence of properly distributed residues capable of forming the stabilizing side-chain ladders (internal or external side-chain stacks, Branden and Tooze, 1999). So far, only the infectious PrP^Sc^ is reported to adopt such a structure (Vázquez-Fernández et al., 2016; Wille and Requena, 2018). **(B)** Formation of the antiparallel β-barrel structures: The register of the initial antiparallel homodimers is determined by the pattern of “strand” propensities since the free energy of backbone-backbone H-bonding, according to the underlying PMO theory, is minimized in the antiparallel alignment by binding backbone segments of *similar* “strand” propensities e.g., C_5_↑C_5_↓ or C_7eq_↑C_7eq_↓, cf. Figure 2. The head-to-tail aggregation of extended conformers of such antiparallel dimers and tetramers is assumed to yield oligomers which by a combination of twist, bend and rise can fold into “jelly-roll”-like cylindrical structures and subsequently into β barrels. Depending on the extension of its core, the initial barrel may convert into a concentric β barrel (Shafrir et al., 2010; Durell et al., 2018); while no such structures were actually isolated, this pathway seems to be consistent with a wide range of indirect evidence (Durell et al., 2018). **(C)** Formation of the parallel cross-β structures: The head-to-tail aggregation of coiled-coil conformers of the antiparallel dimers is assumed to yield circular wedge-shaped paranuclei (Fu et al., 2015; Economou et al., 2016); higher-order annular aggregates of such paranuclei are then expected to undergo conformational antiparallel-to-parallel β structure conversion to yield fibrils which assemble two protofilaments comprising parallel cross-β sheets and aligned in the antiparallel fashion (Schmidt et al., 2015). A collapse of a cross-β sheet onto itself, to form a β arcade, may lead to a separation of the two protofilaments and remodeling of the nascent fibrils into a wide range of alternative assemblies (Tycko, 2015; Colvin et al., 2016; Wälti et al., 2016). The inserted structure diagrams and AFM images are taken from the articles cited in the caption.

According to the PMO theory of misfolding and aggregation of pleiomorphic proteins associated with common brain proteinopathies (Aβ, tau, αS, and PrP^C^), polymerization of the intrinsically disordered amyloidogenic regions of these proteins depends on the distribution of backbone density which determines conformational and H-bonding propensity of the main chain (Cieplak, 2017). The relationship between the backbone polarization and conformational preferences of a given polypeptide chain is here quantified by the *folding potential* function *FP*_*i*_, a function of the NMR shielding tensors of the C^α^ atoms, cf. Computational Methods. Thus, by taking into account the *FP*_*i*_ plots, and conformational properties of β sheets (Salemme, 1983; Branden and Tooze, 1999), one may arrive at a model of polymerization of those proteins. The resulting outline of the anticipated aggregation pathways is indeed shown in Figure 3.

The pathway which would lead to the assembly of tau PHFs is shown in Figure 3C. In the first stage of the process, the accessible segments of the microtubule binding domain MTBD form long antiparallel two-stranded β-sheets, by analogy to the coupled binding and folding od split inteins (Shah et al., 2013; Eryilmaz et al., 2014); the assumed alignment mode is in accord with the results of quantum mechanical modeling of β structure (Cieplak, 2017). In the antiparallel alignment, the free energy of backbone-backbone H-bonding is minimized by binding the MTBD segments of *similar* “strand” propensities e.g., C_5_↑C_5_↓ or C_7eq_↑C_7eq_↓, cf. the model of molecular recognition in formation of β structure in Figure 2. Consequently, the register of the resulting antiparallel tau dimers is determined by the pattern of density distribution and concomitant conformational propensities of the polypeptide backbone of MTBD. The next stage involves head-to-tail association of the antiparallel homodimers into disk-shaped hexamers, and subsequent antiparallel-to-parallel conversion of β structure within the aggregates of such hexamers. This model was previously introduced to account for the rates and morphology of Aβ aggregation on diverse surfaces (Kowalewski and Holtzman, 1999; Qing et al., 2014; Gao et al., 2015), obligatory micelle-like and helical intermediates of Aβ fibrillization (Yong et al., 2002; Roychaudhuri et al., 2009; Vitalis and Caflisch, 2010; Wälti et al., 2015), SAXS data on Aβ dimers (Ryan et al., 2015), AFM data on the morphology of early oligomerization states of Aβ (Fu et al., 2015; Economou et al., 2016), cryo-EM data on the morphology of Aβ fibrils (Schmidt et al., 2015), and the catalysis of fibrillogenesis by the intercalating aromatic ions (Williams et al., 2005; Ladiwala et al., 2011; Bieschke et al., 2012). The presented here outline of divergent pathways of the fibrillization of tau is based on the assumption that this model applies to tau as well. Thus, the assembly of the antiparallel homodimers of tau into hexameric paranuclei—granular aggregates—and their subsequent conversion into the parallel cross-β structure leads to the formation of paired helical filaments. Importantly, the proposed mechanism of this conversion, see the detailed description below, preserves the homodimer's register in the core structure of the nascent fibril.

**Figure 4:**
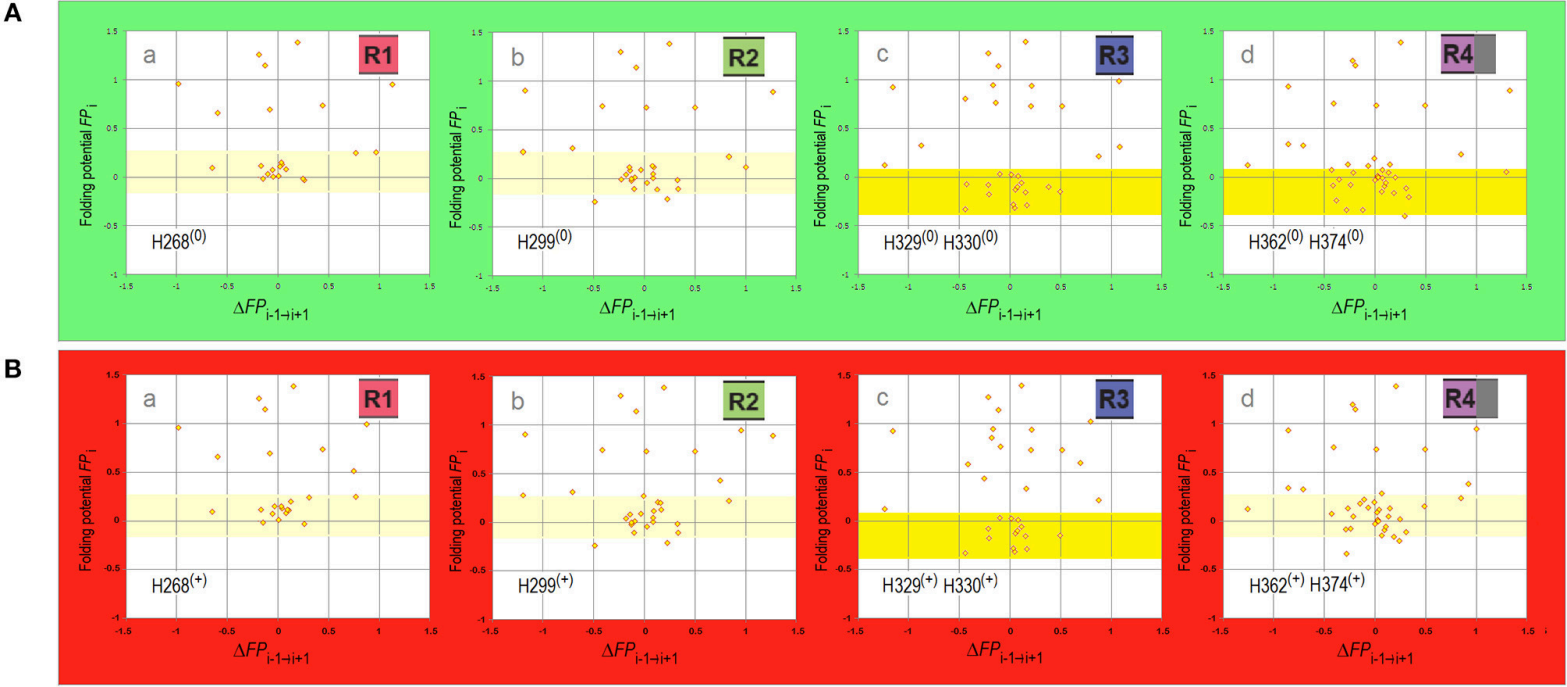
Electronic configuration of the polypeptide backbone and secondary structure propensities of MTBD repeats. **(A)** The *FP*_*i*_ vs. Δ*FP*_*i*−__*1*→*i*+*1*_ plots for MTBD repeats calculated with σ^His^ = −0.2917. The region of *FP*_*i*_ color-coded pale yellow in R1 and R2 corresponds to the ambiguous “strand”/“helix” propensity in water, the region color-coded yellow in R3 and R4 corresponds to “C_5_ strand” propensity in water. (a) Truncated R1, residues 252–274 (omitted the proline-rich segment ^247^PVPMP^251^); (b) R2, residues 275–305; (c) R3, residues 306–336; (d) Appended R4, residues 337–376 (appended ^369^KKIETHKL^376^ segment incorporated in some fibril cores). **(B)** The *FP*_*i*_ vs. Δ*FP*_*i*−__*1*→*i*+*1*_ plots for MTBD repeats calculated with σ^His+^ = 0.2584. The data plotted for the same sequences identified above in **(A)**(a–d), and the *FP*_*i*_ regions color-coded as above, indicating water-bound ambiguous “strand”/“helix” propensity in R1, R2, and R4, and water-bound “C_5_ strand” propensity in R3.

It follows that the repeat structure of the amyloidogenic MTB domain, marked by subtle differences in the electronic configuration of the repeat segments of the main chain, is likely to be one source of the observed diversity of tau-fibril morphology. The differences in conformational propensities and aggregation properties of the four repeats of the MTB domain of tau attracted considerable attention (Perez et al., 1996, 2007; von Bergen et al., 2000; Tokimasa et al., 2005; Naruto et al., 2010; Sogawa et al., 2012, 2014; Lathuillière et al., 2017; Macdonald et al., 2019). To gauge whether and how these differences are indeed related to the differences in backbone polarization, the folding potential *FP*_*i*_ is plotted against its “slope” Δ*FP*_*i*−*1*→*i*+*1*_ separately for each repeat, see the *FP*_*i*_ vs. Δ*FP*_*i*−*1*→*i*+*1*_ plots in Figure 4, where the side chains of the histidine residues of MTBD are either neutral (the side chain's imidazole ring is not protonated and σ^His^ = −0.2917, Table 1), Figure 4A, or cationic (the side chain's imidazole ring is protonated and σ^His+^ = 0.2584, Table 1), Figure 4B. Histidine pK_a_'s vary widely depending on burial within proteins (Edgcomb and Murphy, 2002; Miyagi and Nakazawa, 2008) but the pK_a_ values of the conserved histidines of MTBD are in the physiological range (Charafeddine et al., 2019). It seems safe to assume that depending on the buffer conditions commonly used in the fibrillization experiments (pH 6.0–7.6, anionic cofactors), no histidine side chains are protonated at the upper range of physiological pH, while some or all are likely to be protonated at a lower range of physiological pH. In either case, the scatterplots in Figure 4 are dominated by the presence of multiple “*FP*_*i*_>>0 turns” and Δ*FP*_*i*_≈ 0/Δ*FP*_*i*−*1*→*i*+*1*_≈ 0 clusters, indicating that MTBD lacks backbone elements which would have pronounced secondary structure propensity in aqueous buffers and can therefore adopt “helix” or “strand” fold in a less polar or more polar environment, respectively, cf. Figure 1. On the other hand, however, the “C_5_ strand” propensity is also recognizable, most pronounced in the case of R3 but also in the case of R4, Figure 4Ad, which makes R3 and R4 quite similar in terms of the *FP*_*i*_ profiles when histidines are not protonated. This similarity is lost upon protonation of histidine residues which makes R4 similar in terms of the *FP*_*i*_ profiles to R1 and R2, see Figures 4Ba,b,d.

**Figure 5:**
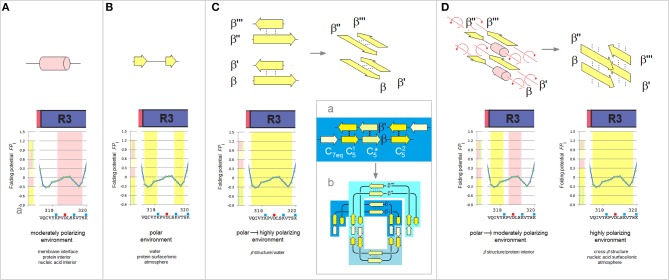
The polarizing effect of the medium, secondary structure propensity of R3, and the mechanism of antiparallel-to-parallel β structure conversion as a path to paired helical filaments of tau. **(A)** The V306-K321segment of the third repeat is expected to fold into a “helix” in a moderately polarizing environment e.g., in the interior of a microtubule complex. The relation between the polarizing effect of the medium and the secondary structure propensity of the polypeptide backbone is indicated in these diagrams by the color-coded bars, cf. Figure 1B, juxtaposed with the *FP*_*i*_ plots. **(B)** The V306-K321 segment is expected to remain disordered in the aqueous buffers, see text. **(C)** The V306-K321 segment is stabilized in water in the “C_5_ strand” configuration by the formation of a two-stranded antiparallel β sheet (binding and folding “gain-of-structure”). The two antiparallel β sheets can in principle be superposed in the parallel alignment as shown in the diagram on the right; (a) The archetypal ‘productive’ homodimer of the amyloidogenic region comprises three β-sheets ${\text{C}}_{5}^{1}↑$${\text{C}}_{5}^{2}↓$/${\text{C}}_{5}^{*}↑$${\text{C}}_{5}^{*}↓$/${\text{C}}_{5}^{2}↑$${\text{C}}_{5}^{1}↓$ and is “appended” by the N-terminal “C_7eq_ strands”; (b) The head-to-tail aggregate of three homodimers folded into a disk-shaped paranucleus. Note the parallel alignment of the two superposed homodimers. **(D)** The V306-K321 segments lose the “C_5_ strand” propensity and become disordered when they are placed in a poorly polarizing environment of the protein interior as a result of aggregation of the paranuclei. The antiparallel β sheets dissociate and the rotation about the strand axes yields parallel alignment of the V306-K321 segments. In the highly polarizing environment of the extended β-cross structure, cf. Figure 1B(d), these segments will again attain the “C_5_ strand” configuration.

Extensive experimental studies point to R3 as crucial to the initiation of PHFs assembly and the following examination focuses on this repeat. The “barbell”-shaped *FP*_*i*_ profile of the V306-K321 segment of R3 shows, Figure 5, two hexapeptide motifs, V306-K311 (PHF6) and S316-K321, which have “C_5_ strand” propensity in the aqueous buffers. The two hexapeptides are however connected by the string of residues P312-K317 with the helical *FP*_*i*_ profile and low *FP*_*i*_ so that in water this string can form neither a stable “turn” nor a stable “helix” and the entire segment remains disordered, Figure 5B. Once transferred, however, into a less polar environment e.g., protein interior, membrane interface or a non-polar solvent, the V306-K321 segment acquires “helix” propensity, Figure 5A cf. Figure 1Ba, and it does indeed become helical in TFE solutions (Minoura et al., 2003; Tokimasa et al., 2005). On the other hand, in a more polarizing environment created by the backbone-backbone H-bonding (Sheridan et al., 1979; Cieplak and Sürmeli, 2004) in a two-stranded antiparallel β sheet formed in water, the entire V306-K321 segment acquires “C_5_ strand” propensity cf. Figure 5C.

The ability to shift secondary structure preference upon the change of environment is essential to tau function but also underlies the “gain-of-structure” process, Figures 5C,D, that ultimately brings about the formation of paired helical filaments. As was mentioned earlier, the segments of MTBD first form long antiparallel two-stranded β-sheets. An example of such a homodimer which comprises three β-sheets ${\text{C}}_{5}^{1}↑$${\text{C}}_{5}^{2}↓$/${\text{C}}_{5}^{*}↑$${\text{C}}_{5}^{*}↓$/${\text{C}}_{5}^{2}↑$${\text{C}}_{5}^{1}↓$ and is “appended” by the N-terminal “C_7eq_ strands,” is shown in Figure 5Ca. The head-to-tail aggregation of those dimers via domain swapping (here interlocking of the N-terminal strands to form the C_7eq_↑C_7eq_↓ β-sheets) yields disk-shaped hexameric polymerization nuclei. The circular conformation of these hexamers superposes the two-stranded antiparallel β-sheets on top of each other in the parallel alignment, see Figure 5Cb.

As the polymerization nuclei subsequently assemble into granular aggregates, the V306-K321 segments are transferred from an aqueous environment into a less polar environment of the protein interior and become again disordered, Figure 5D. Consequently, the antiparallel β-sheets (β*↑β*'↓ and β”↑β”'↓) are destabilized and their strands can rotate about the axes to form the parallel β-sheets (β*↑β*”↑ and β'↑β”'↑) that extend the cross-β structure, see Figure 5D; the highly polarizing environment of the cross-β structure turns the V306-K321 segments back into the “C_5_ strands.” Thus, the nascent fibrils of tau comprise two parallel cross-β sheets (protofilaments) which are aligned in the antiparallel fashion, so that the 2-fold symmetry of the original homodimers is retained; the “appended” strands of the dimers (e.g., C_7eq_ in Figure 5Ca) are not incorporated in the cross-β structure. Collapse of a cross-β sheet onto itself, to form a β arcade, may lead to a separation of the two protofilaments and remodeling of the nascent fibrils into a wide range of alternative assemblies. Regardless, *the core of the tau fibril is produced by conformational conversion of the antiparallel* β *structure of the initial homodimer: the composition of the core reflects the register and extension of the* β *structure of the homodimer*.

**Figure 6:**
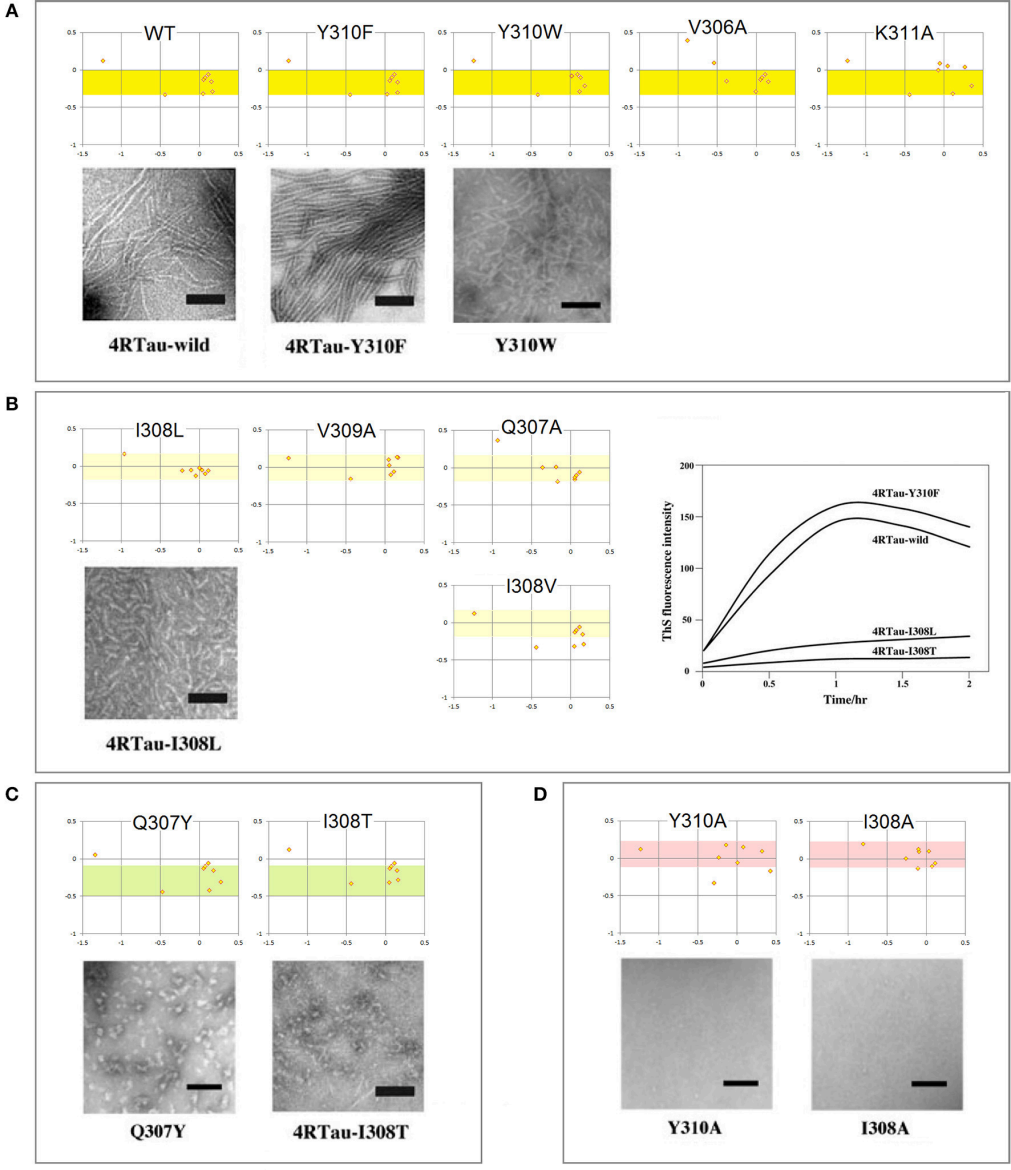
Electronic configuration of the polypeptide backbone and aggregation properties of single-site mutants of tau PHF6: the *FP*_*i*_ vs. Δ*FP*_*i*−__*1*→*i*+*1*_ plots and EM morphology. The results obtained by the heparin-induced fibrillization experiments conducted in Tris-HCl buffer at pH 7.6: **(A)** Mutants forming wild-type filaments. All the mutants retain the “C_5_ strand” propensity of wt PHF6 (the yellow-color coded region of *FP*_*i*_). **(B)** Mutants forming short filaments. The “C_5_ strand” propensity of PHF6 tends to be attenuated compared to the wild type (the pale yellow-color coded region of *FP*_*i*_); the plot on the right-hand side illustrates the effect of mutations on the intensity of thioflavin S fluorescence. **(C)** Mutants forming granules. The “C_5_ strand” propensity of PHF6 tends to be increased compared to the wild type (the green-color coded region of *FP*_*i*_). **(D)** Mutants forming neither granules nor filaments. The “C_5_ strand” propensity of wt PHF6 is lost, replaced by the “helix” propensity (the red-color coded region of *FP*_*i*_). The inserted images are taken from the articles cited in the text.

The mechanism of conformational conversion presented in Figure 5 implies that the fibrillization of tau depends *inter alia* on the balance between the “C_5_ strand” and “helix” propensities of R3. For instance, a significant increase in the “C_5_ strand” propensity may facilitate initial formation of granular aggregates but hinder the subsequent antiparallel-to-parallel β structure conversion. Thus, substitutions, deletions, post-translational modifications etc. in the V306-K321 segment may facilitate or impede fibrillization depending on the effect on this balance. Indeed, replacement of a single residue within PHF6 may (i) modify morphology of filaments, (ii) prevent conversion of granular aggregates into filaments, or (iii) stop aggregation altogether (Naruto et al., 2010; Sogawa et al., 2012, 2014). In agreement with the mechanism in Figure 5, these outcomes tend to be consistent with the changes in the *FP*_*i*_ vs. Δ*FP*_*i*−*1*→*i*+*1*_plots, see Figure 6.

### (ii) Electronic Configuration of the Polypeptide Backbone and the Divergent Pathways of Fibrillization of the 3R MTB Domain: Tau Inclusions in Alzheimer's, CTE and Pick's Disease

The cryo-EM investigation has recently shown that paired helical filaments of tau isolated from the brains of Pick's, Alzheimer's and chronic traumatic encephalopathy (CTE) patients are considerably different in their fibril-core structure (Fitzpatrick et al., 2017; Falcon et al., 2018, 2019). It is reported that only two repeats of 3R-tau (R1-R3R4) are retained in the core of PHFs isolated from the brains of the Alzheimer's and CTE patients, but all three repeats are incorporated in the fibril core of PHFs isolated from the brains of the Pick's patients; besides, the contact between the protofilaments occurs at different register, in the center of the third repeat in Pick's fold, and between the third and fourth repeats in the Alzheimer's fold and CTE fold type II. Here we demonstrate that these differences are consistent with the changes in the pattern of MTBD backbone polarization brought about by the protonation of His268 and His362.

**Figure 7:**
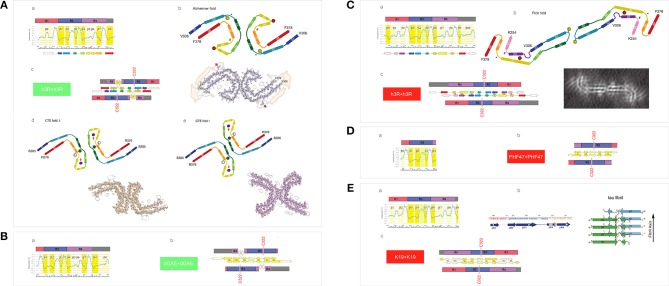
Electronic configuration of the polypeptide backbone and divergent pathways of fibrillization of 3R isoforms: tau inclusions in Alzheimer's, CTE and Pick's disease. **(A)** (a) The *FP*_i_ profile of the tau repeat domain 3R (R1-R3R4), calculated with σ^His^ = −0.2917, indicates “C_5_ strand” propensity of the segments: V306-K311(β1), L315-K321(β2), I328-H330(β3), V337-E342(β4), L357-H362(β7), K370-T377(β8). The *FP*_*i*_ assignment of the secondary-structure propensity is compared to the strand assignment in the Alzheimer's tau fold, see the “rainbow-color coded” bar immediately below the *FP*_*i*_ plot; (b) the Alzheimer-fold diagram, taken from Fitzpatrick et al. (2017); (c) a model of the seeding-competent antiparallel homodimer. Notice that the C322 residues are far apart in this dimer; (d) the CTE type-II, the fold diagram taken from Falcon et al. (2019), which apparently retains the 2-fold symmetry of the nascent fibril. Notice the presence of a hydrophobic co-factor encased (viz. the red arrow) in the protofilament's β arcade; (e) the CTE type-I, the fold diagram taken from Falcon et al. (2019). The structure apparently generated by the interface shift within the CTE type II PHFs. **(B)** (a) The *FP*_i_ profile of the truncated repeat-domain construct dGAE at pH 7.4. In the dGAE construct, the loss of the first repeat is compensated by the addition of a long C-terminal segment immediately following R4; (b) the corresponding model of the homodimer. **(C)** (a) The *FP*_i_ profile of the tau repeat domain 3R (R1-R3R4), calculated with σ^His+^ = 0.2584 for His268 and His362, shows the change from “C_5_ strand” to “C_7eq_ strand” propensity in the segment L357-H362 [β8 vs. β7 in **(A)**(a)] and the enhancement of “C_7eq_ strand” propensity in the segment E263-Q269 [β2 vs. β0 in **(A)**(a)]. The *FP*_*i*_ assignment of the secondary-structure propensity is compared to the strand assignment in the Pick's tau fold, see the ‘rainbow-color coded’ bar immediately below the *FP*_*i*_ plot; (b) the Pick-fold diagram, taken from Falcon et al. (2018); (c) a model of the seeding-competent antiparallel homodimer. Notice that the shift from the “C_5_ strand” to the “C7_eq_ strand” propensity results in the change in register so that the C322 residues are now placed in the dimer in the immediate vicinity of each other. **(D)** (a) The *FP*_i_ profile of the truncated repeat-domain construct HPF47 calculated with σ^His+^ = 0.2584 for His268; (b) the homodimer. **(E)** (a) The *FP*_i_ profile of the truncated repeat-domain construct K19 calculated with σ^His+^ = 0.2584 for His268 and His362. The K19 construct has no C-terminal extension but retains the first repeat; (b) the solid-state NMR assignment of β structure in the fibril core; (c) the corresponding model of the homodimer.

First, we examine the *FP*_*i*_ plot for R1-R3R4 assuming that these two histidine side chains are not protonated (σ^His^ = −0.2917, Table 1), see Figure 7Aa. The *FP*_*i*_-based assignment of the anticipated secondary structure propensities is indeed in accord with the cryo-EM based assignment of β structure in the tau PHFs isolated from the Alzheimer's and CTE brains, see the “rainbow-color coded” bar immediately below the *FP*_*i*_ plot, the Alzheimer-fold diagram on the right-hand side, Figure 7Ab, and the CTE-fold diagrams below, type II and I, Figures 7Ad,e. Note that the CTE isolates are complexes of tau with a hydrophobic cofactor or cofactors; possibly that is why only the minor fraction, type II, retains the 2-fold symmetry of the hypothetical nascent fibril, while the major fraction, type I, lacks this symmetry because of an interface shift.

The expected antiparallel homodimer 3R↑3R↓ is shown in Figure 7Ac. The register of dimerization is controlled by “matching” the anticipated “C_5_ strands” i.e., the R3 and R4 repeats which have similar *FP*_*i*_ profiles under these conditions (cf. Figure 4): β1↑β8↓, β2↑β7↓ and β3↑β4↓. The segments L253-G276 (β0 in R1) and L346-K353 (β5 and β6 in R4) are not incorporated in the β antiparallel structure of the dimer, the first remaining “free,” as the unattached “C_7eq_ strand,” and the other forming a loop. The “free” β0 strand is essential to further aggregation via “domain swapping,” cf. Figure 5Cb, and would not be retained in the fibril core. The 2-fold axis of the anticipated homodimer structure is centered between the repeats R3 and R4, and this register correctly places the C322 residues far apart from each other, cf. Figure 7Ab.

The results of polymerization of the truncated tau construct dGAE corroborate the proposed model. The PHFs assembly of dGAE occurs in 10 mM phosphate buffer, pH 7.4, in the absence of the anionic cofactors (Al-Hilaly et al., 2017). The dGAE construct comprises only two repeats but also, in addition, the C-terminal E372-E394 segment. In this construct, the repeats R3 and R4, and their *FP*_*i*_ profile (σ^His^ = −0.2917), see Figure 7Ba, are identical to those in Figure 7Aa. The repeat R1 is absent but its role as the “free” β0 strand can be assumed here by the C-terminal segment. Thus, the dGAE↑dGAE↓ homodimer is expected to have the same core of antiparallel β structure as the Alzheimer 3R↑3R↓ dimer, and similar capacity for further polymerization via “domain swapping.” The C322s would be far apart in this homodimer, Figure 7Bb, and indeed the assembly of dGAE PHFs is impeded under the non-reducing conditions at pH 7.4.

To consider now an alternative homodimer, we examine the *FP*_*i*_ plot for R1-R3R4 assuming that the side chains of His268 in R1 and His362 in R4 are protonated (σ^His+^ = 0.2584, Table 1), see Figure 7Ca. As shown in Figure 4, the His362(^0^) → His362(^+^) transition considerably alters the *FP*_*i*_ profile of R4 which is now similar to R1 rather than to R3. In fact, the new *FP*_*i*_ assignments of secondary structure propensity readily align with the β structure assignment for the tau PHFs isolated from the Pick's brains, see the “rainbow-color coded” bar below the *FP*_*i*_ plot, and the Pick-fold diagram on the right-hand side, Figure 7Cb. Thus, at the moderately reduced pH, the register of dimerization is controlled by “matching” the anticipated “C_7eq_ strands” in R1 and R4 repeats: β1↑β8↓ and β2↑β7↓; this register is also stabilized by “matching” the “C_5_ strands”: β3↑β6↓ and β4↑β5↓, possibly β0↑β9↓ as well. As a result, all the repeats, including R1, are incorporated in the core of the antiparallel β structure of the homodimer, while the role of the “free” strand is assumed by the C-terminal segment, the β00 strand in the *FP*_*i*_ plot in Figure 7Ca. The 2-fold axis of the dimer is now centered in the middle of the repeat R3 so that the C322 residues are in register, placed in the homodimer very close to each other, Figure 7Cc.

Again, the results of polymerization of the truncated tau constructs, HPF47 and K19, corroborate the proposed model. Fibrillization of HPF47 and the K19 construct R1-R3R4 occurs in 50mM NH_4_Ac buffer at pH 7.0, in the presence of the anionic cofactor heparin, and in both cases polymerization is accelerated under the non-reducing conditions (Friedhoff et al., 1998; von Bergen et al., 2000; Andronesi et al., 2008; Daebel et al., 2012). Assuming that the two histidine side chains are protonated under these conditions (σ^His+^ = 0.2584), the *FP*_*i*_ plot for PHF47 implies that the register of the homodimer does place the C322 residues very close to each other, see Figures 7Da,b. The *FP*_*i*_ plot for K19, Figure 7Ea, shows that the core of the antiparallel β structure will incorporate all three repeats; the N-terminal segment of R1 assumes the role of the “free” β0 strand. The solid-state NMR data indicate that at least a part of R1 is indeed incorporated in the fibril core, Figure 7Eb (Andronesi et al., 2008). In this case as well the C322 residues are placed very close to each other in the homodimer, Figure 7Ec.

These data imply that one possible reason for the divergent fibrillization of tau in Alzheimer's and CTE disease on one hand, and Pick's disease on the other hand, may be the difference in the environment's pH. Interestingly, tau pathology in Alzheimer's initially spreads from entorhinal cortex and locus coeruleus to the hippocampus (Goedert et al., 2017), and there are reports to suggests that the pH in the left hippocampus of Alzheimer's patients does increase rather than decrease in the age-related manner (Mecheri et al., 1997; Mandal et al., 2012; Cichocka et al., 2018). In contrast, the assembly of tau prions in Pick's disease would presumably be facilitated by a moderate drop in pH that accompanies e.g., transit in the endosomal system, inflammation response or an ischemic injury.

### (iii) Electronic Configuration of the Polypeptide Backbone and Divergent Pathways of Fibrillization of 4R Isoforms: Heterogeneity of the Heparin-Induced Tau Fibrils and Asymmetric Cross-Seeding Barriers

**Figure 8:**
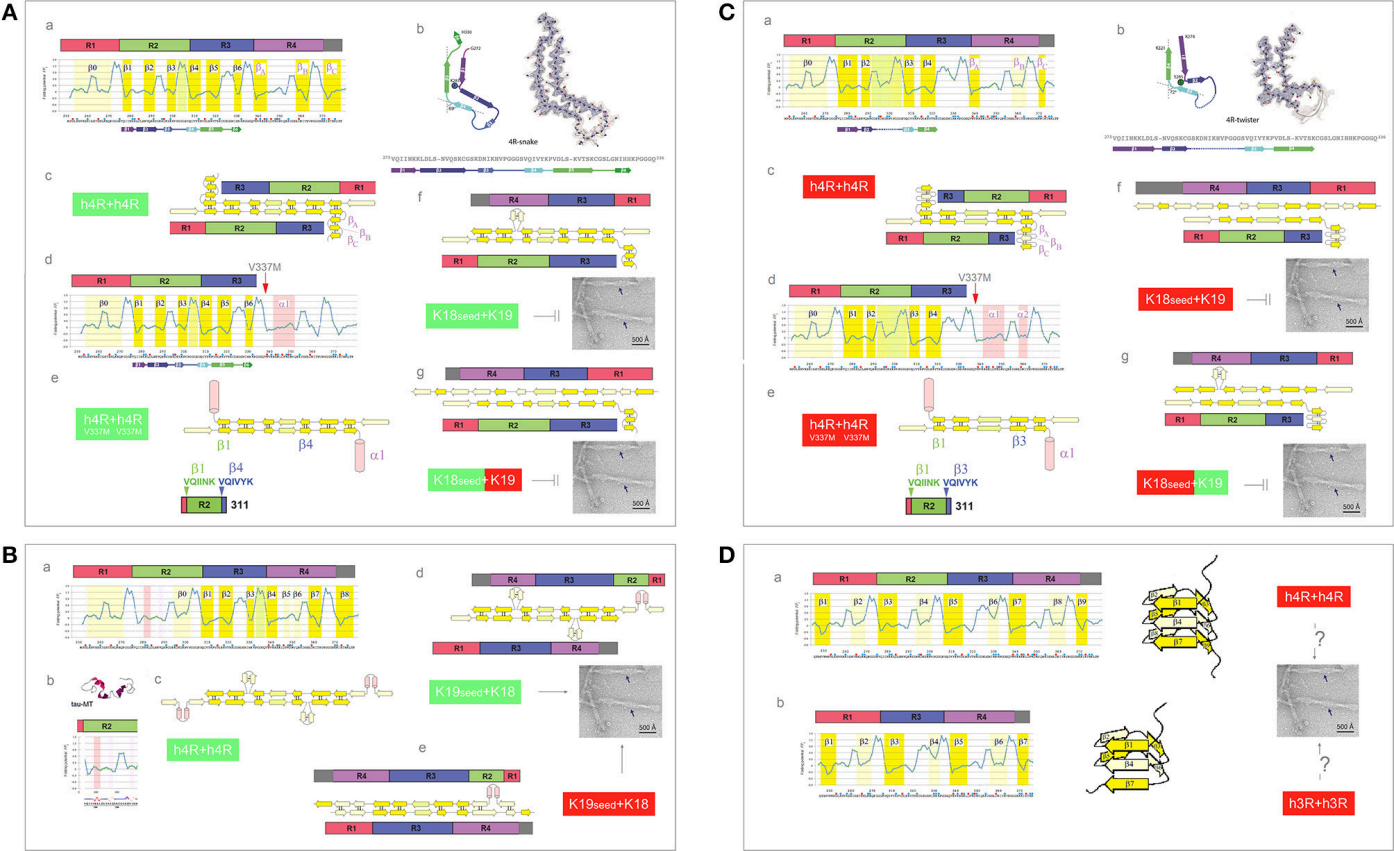
Electronic configuration of the polypeptide backbone and divergent pathways of fibrillization of 4R isoforms: heterogeneity of the heparin-induced tau fibrils and asymmetric cross-seeding barriers. **(A)** (a) The *FP*_i_ profile of the tau repeat domain 4R (R1R2R3R4) calculated with σ^His^ = −0.2917. The *FP*_*i*_ assignment of the secondary-structure propensity is compared to the strand assignment in the 4R-snake fold of tau fibrils, see the color-coded bar immediately below the *FP*_*i*_ plot; (b) The diagram of the 4R-snake fold, taken from Zhang et al. (2018); (c) The corresponding model of the staggered antiparallel homodimer 4R↑4R↓; (d) The *FP*_i_ profile of the repeat domain 4R (R1R2R3R4) of the tau V337M. Note the change of the propensity of R4 into “helix”; (e) The corresponding model of the antiparallel homodimer 4R↑4R↓ with R4 folded into “helix”; (f) The model of the antiparallel “non-protonated” heterodimer 4R↑3R↓, notice the lack of 2-fold symmetry. The model implies that the K18 seeds cannot template oligomerization of K19 under these conditions; (g) The model of the antiparallel 3R(^+^)↓4R↑ heterodimer, notice the lack of 2-fold symmetry. The model implies that K18 seeds cannot template oligomerization of K19 under these conditions either. **(B)** (a) The *FP*_i_ profile of the tau repeat domain 4R (R1R2R3R4) calculated with σ^His+^ = 0.2584 for H299, and selectively induced into “helix” in R2; (b) The *FP*_i_ profile of R2 bound to microtubule as a “helix” bundle, PDB ID 2mz7; (c) The corresponding model of the staggered antiparallel homodimer 4R↑4R↓; (d) The model of the alternative “non-protonated” 3R↑4R↓ heterodimer. Notice that this heterodimer is seeding-competent according to the underlying PMO theory, Figure 5, which implies that the K19 seeds can template oligomerization of K18; (e) The model of the alternative “protonated” 3R↑4R↓ heterodimer. Notice that this heterodimer is seeding-competent according to the underlying PMO theory, Figure 5, which implies that the K19 seeds can template oligomerization of K18. **(C)** (a) The *FP*_i_ profile of the tau repeat domain 4R (R1R2R3R4) calculated with σ^His+^ = 0.2584. The *FP*_*i*_ assignment of the secondary-structure propensity is compared to the strand assignment in the 4R-twister fold of tau fibrils, see the color-coded bar immediately below the *FP*_*i*_ plot; (b) The diagram of the 4R-twister fold taken from Zhang et al. (2018); (c) The corresponding model of the staggered antiparallel homodimer 4R↑4R↓; (d) The *FP*_i_ profile of the repeat domain 4R (R1R2R3R4) of the tau mutant V337M. Notice the change of the propensity of 4R into “helix”; (e) The corresponding model of the antiparallel homodimer 4R↑4R↓ with R4 folded into “helix”; (f) The model of the protonated 3R↑4R↓ heterodimer. Notice that this heterodimer is *not* seeding-competent according to the underlying PMO theory, Figure 5, which implies that the “protonated” K18 seeds cannot template oligomerization of K19 under these conditions; (g) The model of the 4R(^+^)↑3R↓ heterodimer. Notice that this heterodimer is *not* seeding-competent according to the underlying PMO theory, Figure 5, which implies that the “protonated” K18 seeds cannot template oligomerization of K19 under these conditions either. **(D)** The *FP*_i_ plots illustrate the hypothetical patterns of “strand” propensities produced by protonation of MTBD histidines (σ^His+^ = 0.2584) which would be consistent with folding of MTBD into a β helix (cf. Figure 3A). In contrast to the antiparallel alignment, the parallel alignment of a two-stranded β sheet is stabilized, according to the underlying PMO theory see Figure 2, by the backbone-backbone H-bonding between two segments of *different* “strand” propensities e.g., C_7eq_↑C_5_↑. (a) 4R-tau isoform, the diagram shows the corresponding right-handed three-rung β solenoid; (b) 3R-tau isoform, the diagram shows the corresponding right-handed two-rung β solenoid.

The presence of an additional repeat segment in the 4R-tau isoforms increases the complexity of the aggregation pathways and raises the issue of co-aggregation and cross-seeding barriers. The recent cryo-EM investigation reveals that the heparin-induced fibrillization of 4R-tau in 30 mM MOPS at pH 7.2 (1 mM AEBSF, with 4 mM TCEP) yields a heterogeneous mixture of filaments formed by the second and third repeats only (Zhang et al., 2018). The plausible homodimer structures which account for the two most abundant PHFs are shown in Figures 8A,C. The first *FP*_*i*_ plot (Figure 8Aa, σ^His^ = −0.2917) correctly anticipates the six β strands found in the 4R-snake fibrils, Figure 8Ab. The fourth repeat is assumed to remain outside of the β structure of the homodimer, possibly folded into a β-sheet meander, Figure 8Ac, or into a helix comprising the K340-K353 segment of R4; such a helix would be stabilized by the V337M mutation associated with the familial FTLD, see Figure 8Ad. In any event, the 2-fold axis of the homodimer would be centered between the repeats R2 and R3 and only those two repeats would be incorporated in the fibril core, Figures 8Ac,e.

So far, there is no report of the 4R fibrils that incorporate the R3-R4 rather than the R2-R3 repeats. The *FP*_*i*_ profile of the R2 repeat suggests however that the register of the 4R-tau homodimers can be redirected when a non-polar environment stabilizes helical conformations of the N279-Q288 segment, in particular when H299 is protonated, see Figures 8Ba–c. Thus, some selective interactions with the protein, lipid, polyanionic cofactors or the membrane interface, or even selective polarizing or depolarizing interactions with other segments of tau molecule, could trigger such an alternative fibrillization pathway.

Presently, the reason for the difference in the register of 3R-tau dimers and heparin-induced 4R-tau dimers cannot be ascertained. One may note however that the free energy of backbone-backbone H-bonding could be minimized in the (R2-R3)↑(R2-R3)↓ complex rather than in the (R3-R4)↑(R3-R4)↓ complex, in spite of the more ambiguous “C_5_ strand” in-water-propensity of R2 compared to R4, because of the more advantageous spacing of its “strand” segments. If this is indeed the case, it follows that the asymmetric barrier to cross-seeding of the truncated constructs K18 and K19 could result from the differences in backbone polarization of the MTBD repeats. Assuming that the “non-protonated” truncated 4R-tau construct K18 adopts the same configuration as the one shown in Figure 8A, K18 seeds are not expected to induce fibrillization of the truncated 3R-tau construct K19: the hypothetical heterodimers lack the 2-fold symmetry and would not be stable enough to be “productive,” see Figures 8Af,g. On the other hand, if the “non-protonated” K19 construct adopts the configuration as the one shown in Figure 7A, K19 seeds can template oligomerization of K18, see Figure 8Bd; this might even be true for the “protonated” K19, Figure 8Be. Regardless, it seems that the 3R and 4R isoforms could co-aggregate under the conditions where His268 and His362 side chains remain neutral.

One plausible way to account for the second most abundant PHF in the heparin-induced fibril mixture, 4R-twister, is to consider protonation of MTBD histidines. The *FP*_*i*_ plot (Figure 8Ca, σ^His+^ = 0.2584) does indeed correctly anticipate the four strands found in the “4R-twister” fibrils, Figure 8Cb. The register of the homodimer is now shifted, the 2-fold axis is now centered in the middle of the repeat R2, while the repeat R4 is likely to fold into a coil or a helix, outside of the antiparallel β structure of the dimer, Figures 8Cc–e. Note that the “helix” fold of the K340-K353 segment can also be stabilized by further drop in pH and Glu^−^ → *Glu*^0^ protonation (Cieplak, 2017). As for the experimental evidence of fibrillization at low pH, 4R-tau, and K18 were reported to form fibrils at pH 6.0 in the presence of polyglutamates (Nizynski et al., 2018), and aggregation of 4R-tau was reported to be slowed down in acetic acid buffer at pH 4.5 (Nishiura et al., 2010), while K19 is reported not to aggregate at pH 2.0 (Andronesi et al., 2008). As shown in the diagrams in Figures 8Cd,e, the “protonated” 4R↑3R↓ heterodimers lack the 2-fold symmetry and would not be stable enough to be “productive”; accordingly, the 3R and 4R isoforms would not co-aggregate under these conditions.

Lastly, the *FP*_*i*_ plots for both isoforms protonated on all histidines (Figure 8D, σ^His+^ = 0.2584) suggest the emergence of the pattern of alternating “C_7eq_ strand” and “C_5_ strand” propensities of MTBD. Hypothetically, this pattern is expected to facilitate formation of a β solenoid: in the parallel alignment of a two-stranded β sheet, the free energy of backbone-backbone H-bonding is minimized by binding the MTBD segments of *contrasting* “strand” propensities e.g., C_7eq_↑C_5_↑, cf. Figures 2, 3A.

### (iv) Electronic Configuration of the Polypeptide Backbone and the Aggregation Properties of the MAP Homologs

**Figure 9:**
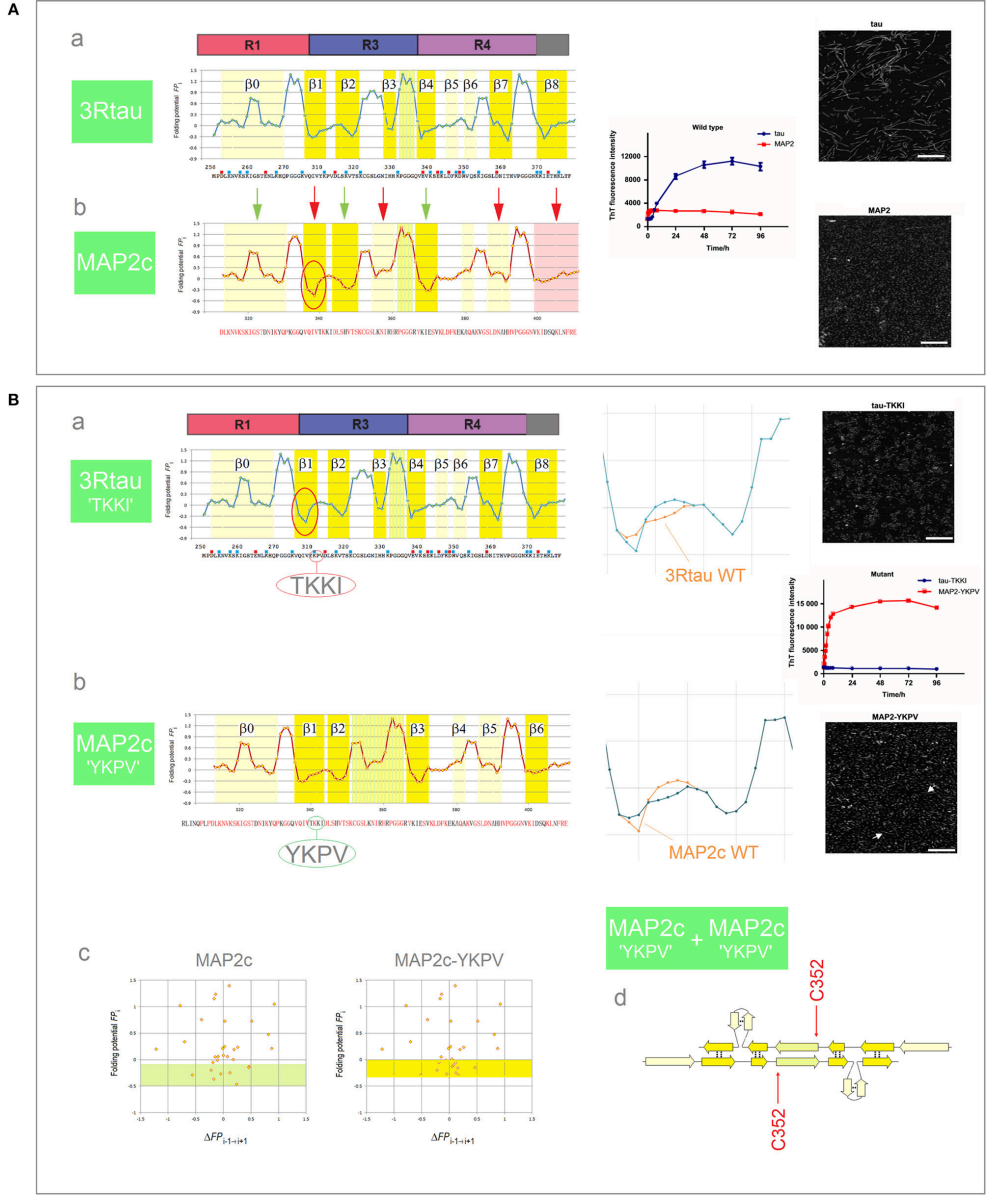
Electronic configuration of the polypeptide backbone and the aggregation properties of MAP homologs. **(A)** (a) The *FP*_*i*_ profile of the 3R (R1-R3R4) isoform of tau and the anticipated secondary structure propensity in water. The adjacent plot of the ThT fluorescence intensity vs. time (blue curve) and the insert show the assembly of PHFs; (b) The *FP*_*i*_ profile of MAP2c and the anticipated secondary structure propensity in water. The adjacent plot (red curve) and the insert show that MAP2c does not form PHFs, polymerization stops at the stage of granular aggregates. The green and red arrows point to the retained and altered, respectively, secondary structure assignments; note the marked increase in the “C_5_ strand” propensity in the segment corresponding to the V306-K321 segment of R3, see the text. **(B)** (a) The *FP*_*i*_ plot for 3R tau-TKKI mutant, see the text, and the anticipated secondary structure propensity in water. The right-hand diagram compares the superposed *FP*_*i*_ profiles of PHF6 in WT and mutant protein. The adjacent plot of the ThT fluorescence intensity vs. time (blue curve) and the insert show that the mutant does not form PHFs; (b) The *FP*_*i*_ plot for MAP2c-YKPV mutant, see the text, and the anticipated secondary structure propensity in water. The right-hand diagram compares the superposed *FP*_*i*_ profiles of the PHF6-like segment in WT and mutant protein. The adjacent plot of ThT fluorescence intensity vs. time (red curve) and the insert show the assembly of fibrils, viz. the white arrows in the insert; (c) The *FP*_*i*_ vs. Δ*FP*_*i*−*1*→*i*+*1*_ plots for MAP2c and the MAP2c-YKPV mutant, note the shift in the “C_5_ strand” propensity, up from the *FP*_*i*_ region characteristic for PHF6 mutants that form granular aggregates, Figure 6C, to the region characteristic for PHF6 mutants that form wild-type filaments, Figure 6A; (d) The anticipated “productive” homodimer of the MAP2c-YKPV mutant.

Tau belongs to the family of homologous microtubule-associated proteins, the closest homolog being MAP2c which, like tau, also forms granular aggregates. In contrast to tau, however, granular aggregates of MAP2c do not convert into fibrils. As was mentioned earlier in section (i), Figure 5, granular aggregates of tau may also lose the capacity to convert into fibrils as a result of a single-site mutation in the PHF6 segment. Interestingly, the *FP*_*i*_ plots point in both cases to the same underlying effect. The MTB domain of MAP2c comprises three repeats and so its *FP*_*i*_ profile can be directly compared to the *FP*_*i*_ profile of the 3R (R1-R3R4) isoform of tau, see Figure 9A. In view of the preceding discussion, one difference stands out amongst overall similarity of the two profiles, see the segment of MAP2c highlighted in red, Figure 9Ab: in place of the ^311^YKPV^314^ fragment of the third repeat of tau, the corresponding fragment in MAP2c has the sequence ^340^TKKI^343^. The replacement lowers the minimum of *FP*_*i*_ and removes the “strand”-perturbing Pro, and the two changes together significantly increase the “C_5_ strand” propensity in water of the entire segment. As described in section (i), Figure 5, such an increase in the “C_5_ strand” propensity may facilitate initial formation of granular aggregates but hinder the subsequent antiparallel-to-parallel β structure conversion. Accordingly, MAP2c forms granular aggregates but not fibrils (Xie et al., 2015), just like the PHF6 mutants of 3R-tau Q307Y and I308T which are also characterized by the increase in the “C_5_ strand” propensity compared to the wild type, and form granular aggregates but not fibrils, cf. Figure 6.

It is sufficient, it turns out, to exchange the YKPV and TKKI tetrapeptides in the sequences of tau and MAP2c in order to completely reverse the aggregation properties of the two proteins: the 3R tau-TKKI mutant forms granular aggregates which do not convert into fibrils, while the MAP2c-YKPV mutant assembles into short fibrils, see Figure 9B (Xie et al., 2015). The change in “strand” propensity due to mutation is highlighted in the diagrams comparing the superposed *FP*_*i*_ profiles of the wild type and mutant proteins; this change is less obvious in the *FP*_*i*_ vs. Δ*FP*_*i*−*1*→*i*+*1*_ plots, Figure 9Bc. The *FP*_*i*_ plot for the MAP2c-YKPV mutant suggests however that the initial “productive” homodimer, Figure 9Bd, would have somewhat different register than the 3R tau homodimer.

## Concluding Remarks

The model of head-to-tail association of the antiparallel homodimers into disk-shaped hexamers, and subsequent antiparallel-to-parallel conversion of β structure within the aggregates of such hexamers, cf. Figure 3, was previously introduced (Cieplak, 2017) to account for the rates and morphology of Aβ aggregation on diverse surfaces (Kowalewski and Holtzman, 1999; Qing et al., 2014; Gao et al., 2015), obligatory micelle-like and helical intermediates of Aβ fibrillization (Yong et al., 2002; Roychaudhuri et al., 2009; Vitalis and Caflisch, 2010; Wälti et al., 2015), SAXS data on Aβ dimers (Ryan et al., 2015), AFM data on the morphology of early oligomerization states of Aβ (Fu et al., 2015; Economou et al., 2016), cryo-EM data on the morphology of Aβ fibrils (Schmidt et al., 2015), and the catalysis of fibrillogenesis by the intercalating aromatic ions (Williams et al., 2005; Ladiwala et al., 2011; Bieschke et al., 2012). The presented here outline of divergent pathways of the fibrillization of tau is based on the assumption that this model applies to tau as well. The reported recently broad diversity of the tau-fibril morphology (Fitzpatrick et al., 2017; Falcon et al., 2018, 2019; Zhang et al., 2018) is thus attributed to the variation in the register of the initial homodimers, a consequence of the variation in backbone polarization of the amyloidogenic region of tau which is determined by examination of the folding potential *FP*_*i*_ profiles. The sources of these variations are the very features which enable tau to perform its function: (i) the repeat structure of the amyloidogenic MTB domain marked by subtle differences in the electronic configuration of the repeats' main chain; (ii) ambiguous conformational propensities of the water-bound amyloidogenic region which make the fold of each separate segment of this region sensitive to the changes in polarity of the medium and molecular embedding i.e., to the selective binding of proteins, lipids, polyanionic cofactors, and selective polarizing or depolarizing interactions with other segments of the tau molecule; and (iii) the conserved His residues with the pK_a_ values in the physiological range (Charafeddine et al., 2019) which make conformational propensity of MTBD sensitive to a moderate drop in pH that accompanies for instance transit in the endosomal system, inflammation response or an ischemic injury.

Surprisingly, this account appears to capture major aspects of morphological diversity in tau fibrillization. It is surprising because the folding potential function *FP*_*i*_ focuses solely on the conformational and H-bonding propensity of the polypeptide backbone, ignoring side chain-side chain interactions. In fact, it would clearly be useful on some occasions to complement the *FP*_*i*_ plots by showing the distribution of the ionized side chains and potential “steric zipper” segments or the presence of proline and cysteine residues in the sequence. Thus, we do not comment in this paper on the posttranslational modifications and single-site mutations which alter MTBD charge or constrain main-chain geometry. Nonetheless, the outcome of the present investigation does suggest that the interactions dependent on backbone density distribution play an important role in conformational behavior of MTBD. This conclusion is in line with the arguments of the backbone-based theory of protein folding (Rose et al., 2006), and with the notion of common origin of amyloidogenicity of proteins associated with major brain proteinopathies.

## Author Contributions

The author confirms being the sole contributor of this work and has approved it for publication.

### Conflict of Interest Statement

The author declares that the research was conducted in the absence of any commercial or financial relationships that could be construed as a potential conflict of interest.
